# Increase in the Complement Activation Product C4d and the Terminal Complement Complex sC5b-9 Is Associated with Disease Severity and a Fatal Outcome in Necrotizing Soft-Tissue Infection

**DOI:** 10.1159/000520496

**Published:** 2021-12-14

**Authors:** Morten Hedetoft, Martin Bruun Madsen, Cecilie Bo Hansen, Ole Hyldegaard, Peter Garred

**Affiliations:** ^a^Department of Anaesthesia, Rigshospitalet, University of Copenhagen, Copenhagen, Denmark; ^b^Department of Intensive Care, Rigshospitalet, University of Copenhagen, Copenhagen, Denmark; ^c^Department of Clinical Immunology Section 7631, Laboratory of Molecular Medicine, Rigshospitalet, University of Copenhagen, Copenhagen, Denmark; ^d^Department of Anaesthesia, Rigshospitalet, University of Copenhagen, Copenhagen, Denmark; ^e^Department of Clinical Immunology Section 7631, Laboratory of Molecular Medicine, Rigshospitalet, University of Copenhagen, Copenhagen, Denmark

**Keywords:** Necrotizing soft-tissue infection, Complement, C4d, Terminal complement complex, Sepsis

## Abstract

The hyperinflammatory burden is immense in necrotizing soft-tissue infection (NSTI). The complement system is a key during the innate immune response and may be a promising target to reduce the inflammatory response, potentially improving the clinical outcome. However, complement activation and its association to disease severity and survival remain unknown in NSTI. Therefore, we prospectively enrolled patients with NSTI and sampled blood at admission and once daily for the following 3 days. Plasma C4c, C4d, C3bc, and C3dg and the terminal complement complex (TCC) were evaluated using ELISA techniques. In total, 242 patients were included with a median age of 62 years, with a 60% male predominance. All-cause 30-day mortality was 17% (95% confidence interval [CI] 13–23) with a follow-up of >98%. C4c and C3dg were negatively correlated with Simplified Acute Physiology Score II (*Rho* −0.22, *p* < 0.001 and *Rho* −0.17, *p* = 0.01). Patients with septic shock (*n* = 114, 47%) had higher levels of baseline TCC than those in non-shock patients (18 vs. 14, *p* < 0.001). TCC correlated with the Sequential Organ Failure Assessment (SOFA) score (*Rho* 0.19, *p* = 0.004). In multivariate Cox regression analysis (adjusted for age, sex, comorbidity, and SOFA score), high baseline C4d (>20 ng/mL) and the combination of high C4d and TCC (>31 arbitrary units/mL) were associated with increased 30-day mortality (hazard ratio [HR] 3.26, 95% CI 1.56–6.81 and HR 5.12, 95% CI 2.15–12.23, respectively). High levels of both C4d and TCC demonstrated a negative predictive value of 0.87. In conclusion, we found that in patients with NSTI, complement activation correlated with the severity of the disease. High baseline C4d and combination of high C4d and TCC are associated with increased 30-day mortality. Low baseline C4d or TCC indicates a higher probability of survival.

## Introduction

Necrotizing soft-tissue infection (NSTI) is a rapidly progressing bacterial infection, causing widespread necrosis in any layer of the soft-tissue compartment [1]. Patients with NSTI are severely ill, and the majority require intensive care, inotropes, and mechanical ventilation [2]. The clinical condition may quickly advance into septic shock and multiple organ failure, and death occurs in approximately 25% within 90 days [3]. NSTI is a rare disease affecting approximately 2 inhabitants per 100,000/year [3, 4]. Early recognition is a key in NSTI, but misdiagnosis has been reported as a problem [5]. Although timely diagnosis and radical and intensive multidisciplinary treatment are achieved, the burden of disease is substantial.

Biomarkers could provide essential information in treatment guidance and decision-making in patients with NSTI [6]. It would be of clinical relevance if biomarkers could identify NSTI patients at high-risk of death as these may benefit from more aggressive treatment. In contrast, low-risk patients could avoid some of the multiple extensive surgical exploration and debridements currently leading to functional limitations in one-third of all NSTI survivors [7]. However, prognostic biomarkers have only been assessed in few observational studies, and none have provided enough robustness to be applied in the clinic [8, 9, 10, 11, 12].

Biomarkers of the complement system may provide valuable prognostic information in NSTI. The complement system is key during the innate immune response, including inflammation and pathogen killing and removal [13]. However, it also contributes to bridging the innate immune and adaptive immune responses [14]. Although the complement system is crucial in the first-line of defence in sepsis, it may well promote an uncontrolled hyper-inflammation in the later phase of sepsis, leading to tissue damage, multiple organ failure, and increased mortality [15, 16, 17]. As the inflammatory response is excessive in NSTI [18], the complement system may well be a promising target to reduce harmful inflammation, potentially improving the clinical outcome. Of particular interest in this regard, we have earlier demonstrated the lectin complement pathway to be of particular relevance in complement activation during NSTI as ficolin-2 − an important pattern recognition molecule used in the activation of the lectin pathway − is associated with both short- and long-term mortality [19]. Furthermore, in 135 patients with NSTI, high C4c/C4 ratio, C3bc, and C3bc/C3 ratio have been associated with increased mortality, whereas high MASP-1 and C4 were associated with improved survival [20]. These results indicate the complement system to be of significant importance for further investigation in patients with NSTI. Increased knowledge of the pathophysiological mechanisms in NSTI may result in a more individualized treatment regime, potentially reducing the disease burden and improving survival. Here, we aimed to evaluate the complement activation products C4c, C4d, C3bc, and C3dg and the fluid phase analogue of the terminal complement complex (TCC) sC5b-9 at admission and once daily during the first 72 h of hospitalization in patients with NSTI and associate the findings with disease severity and survival.

## Materials and Methods

The present study was based on data of the Danish fraction from the INFECT study (ClinicalTrials.gov number; NCT01790698): a prospective, international, multicenter observational study enrolling adult patients with surgically confirmed NSTI to Rigshospitalet (University of Copenhagen, Denmark) between February 2013 and March 2017, in which some data have been reported elsewhere [2, 19, 20].

### Patient Management

All patients were treated with our standardized multidisciplinary treatment protocol comprehensively described elsewhere [2]. In short, all patients were planned to receive frequent surgical debridement (3 revisions during the first 24 h, thereafter repeated as necessary), initial broad-spectrum antibiotics (meropenem, ciprofloxacin, and clindamycin), supportive intensive care, and HBO_2_ treatment (3 sessions of 90 min at 284 kPa within the first 72 h from admission, preferably the first 2 sessions within 24 h).

### Data Collection

Patients had blood sampled into ethylenediaminetetraacetic acid sample tubes upon admission − which was often after initial surgery (baseline) at each of the following 3 days (all between 08:00 and 12:00). Blood samples were centrifuged within 40 min from sampling at 3,500 rpm (2,400 *g*) for 10 min. The plasma was collected and put in cryotubes and stored at −80°C until analysis. Predefined clinical data (e.g., patients' characteristics, biochemistry, microbiological findings, supportive modalities, and outcomes) were registered by dedicated personnel.

### Complement Analyses

Analyses of complement activation products were performed using in-house established ELISA-based techniques and presented as arbitrary units (AU) as described for C4c, C3bc, C3dg, and TCC [21, 22, 23, 24] and in brief below. C4d was measured using a commercially available ELISA from SVAR (COMPL C4d RUO, Malmø, Sweden) according to manufacturer's instructions.

#### C4c ELISA

Nunc MaxiSorp 384-well plates (Thermo Scientific Nunc, Roskilde, Denmark) were coated with 2 μg/mL anti-C4c mouse mAb 99-72-18 in phosphate-buffered saline (PBS) overnight (ON) at 4°C. Following 3 times wash in PBS and 0.05% Tween 20 (PBS-T, Merck, Darmstadt, Germany), plates were blocked for 15 min with PBS-T. Plasma samples diluted 1:300 in PBS-T + 10 mM ethylenediaminetetraacetic acid +0.1% mouse serum +0.5% bovine serum (dilution buffer) were applied to the plates and incubated for 60 min shaking at room temperature (RT). Afterwards, the plates were incubated for 90 min shaking at RT with 0.4 μg/mL polyclonal rabbit anti-human C4c (Agilent/DAKO Technologies, Santa Clara, CA, USA) in PBS-T. After, HRP-conjugated polyclonal swine-anti-rabbit ab (Agilent/DAKO Technologies) diluted 1:2,000 in PBS-T was added and incubated for 60 min shaking at RT.

#### C3bc ELISA

Nunc MaxiSorp 384-well plates were coated with 2 μg/mL mouse anti-C3bc (clone BH6) in PBS ON 4°C. Plasma samples were added to the plates 1:2,000 in dilution buffer and incubated 60 min non-shaking at 4°C. After incubation and washing in PBS-T, 1 μg/mL of polyclonal rabbit anti-human C3c (Agilent/DAKO Technologies) in PBS-T was added to the plates and incubated for 90 min shaking at RT. Afterwards, HRP-conjugated polyclonal swine-anti-rabbit ab (Agilent/DAKO Technologies) diluted 1:2,000 in PBS-T was added and incubated shaking at RT for 60 min.

#### C3dg ELISA

Nunc MaxiSorp 384-well plates were coated with 5 μg/mL mAb 15-36-06 rat-anti-human C3d and incubated ON at 4°C. Plasma samples were diluted 1:50 in dilution buffer, applied to plates, and incubated for 60 min at 20°C. Subsequently, polyclonal anti-human C3d rabbit antibody purchased from Agilent/DAKO Technologies biotinylated in-house diluted 1:1,000 in PBS-T was added and incubated for 60 min at 20°C. Afterwards, HRP-conjugated streptavidin (Zymed, Invitrogen) was added in 1:3,000 dilution and incubated at 20°C for 30 min at RT.

#### TCC ELISA

Nunc MaxiSorp 384-well plates were coated with 2 μg/mL mouse anti-C9 (clone aE11) in PBS ON 4°C. Plasma samples were added to the plates 1:5 in dilution buffer and incubated 90 min shaking at RT. After incubation and washing in PBS-T, 2 μg/mL biotinylated mouse mAb anti-C6 (clone 9C4) in PBS-T was added to the plates and incubated for 90 min shaking at RT. Afterwards, HRP-conjugated streptavidin (Merck) diluted 1:2,000 in PBS-T was added and incubated shaking at RT for 60 min.

Plates were washed in PBS-T between every step. All in-house ELISA assays were developed using tetramethylbenzidine (1 substrate (Kem-En-Tec, Taastrup, Denmark), and the reaction was stopped using 0.2 M H_2_SO_4_. A Synergy HT absorbance reader was used to measure the optical density at 450–630 nm (BioTek Instruments, Winooski, VT, USA).

### Outcomes

The primary analysis was the association between baseline complement levels and 30-day mortality. Secondary analyses included associations between baseline complement levels and disease severity and differences in complement levels according to patients presenting with shock, Group A *Streptococcus*, receiving renal-replacement therapy, or amputation within day 7.

### Statistics

Continuous data are presented as medians with interquartile range (IQR), and categorical data presented as absolute numbers with percentages (%). The Shapiro-Wilk test showed that data were not normally distributed. Consequently, continuous data were compared using the Wilcoxon rank-sum test. Spearman's rank correlation test assessed the correlations. Receiver operating characteristic (ROC) curves were computed and analysed for the area under the curve (AUC). The Youden Index optimal cut-off point was used to categorize low and high complement levels at admission. Prognostic accuracy in predicting the 30-day mortality was addressed by sensitivity, specificity, positive predictive value, and negative predictive value. The log-rank test was used for assessing the difference in the 30-day mortality according to the optimal cut-off level, and multivariate Cox analysis was used to evaluate the association of high/low complement levels and 30-day mortality. The Cox regression results were reported with hazard ratio (HR) and 95% confidence interval adjusted for differences in age, sex, comorbidity (dichotomized; yes/no), and the Sequential Organ Failure Assessment (SOFA) score day 1. *p* values were reported as exact, unless <0.001. *p* values below 0.05 were considered statistically significant. Statistical analyses were performed using Rstudio v. 1.0.136 (RStudio, Inc.) and GraphPad Prism 8.0.2 (GraphPad Inc., San Diego, CA, USA).

## Results

**Fig. 1:**
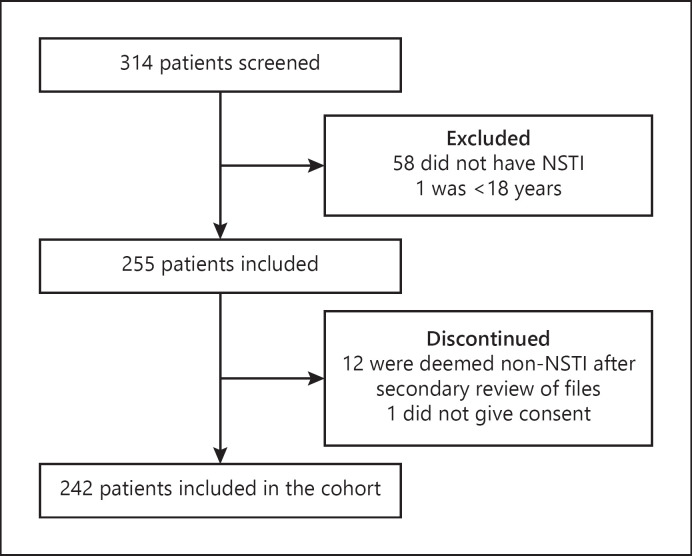
Flow chart of patients included in the study. Patients with suspected NSTI were screened for eligibility. Patients were excluded if they did not meet the criteria of inclusion. After inclusion, patients' files were reviewed, and 12 were deemed non-NSTI due to no intraoperative signs of NSTI. One patient was discontinued as informed consent was not obtainable. NSTI, necrotizing soft-tissue infection.

Two hundred and forty-two patients with NSTI were included between February 2013 and March 2017 (Fig. 1). Patients' characteristics, including baseline complement levels, laboratory values, microbial findings, clinical severity scores, and outcomes, are presented in Table 1. Three patients were lost to the follow-up at day 30 (98.8% follow-up).

### Complement Activation and the 30-Day Mortality

**Fig. 2:**
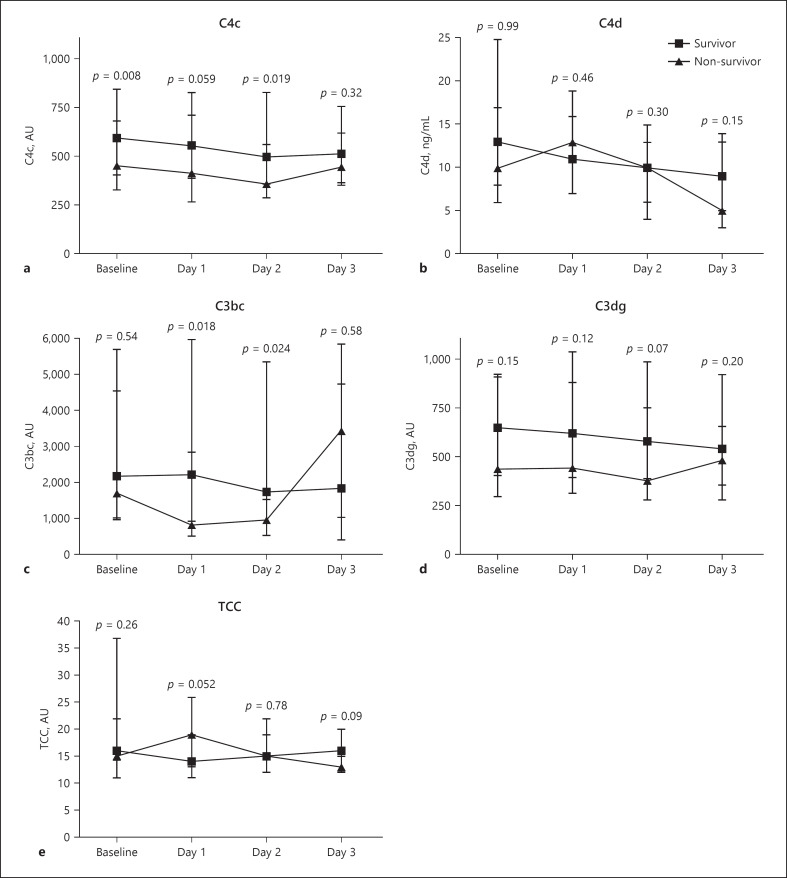
Concentrations of C4c (**a**), C4d (**b**), C3bc (**c**), C3dg (**d**) and TCC (**e**) at admission, day 1, day 2, and day 3 according to survivor and non-survivors. Data are plotted as medians with an IQR. Comparisons were performed by the Wilcoxon rank-sum test. IQR, interquartile range.

**Fig. 3:**
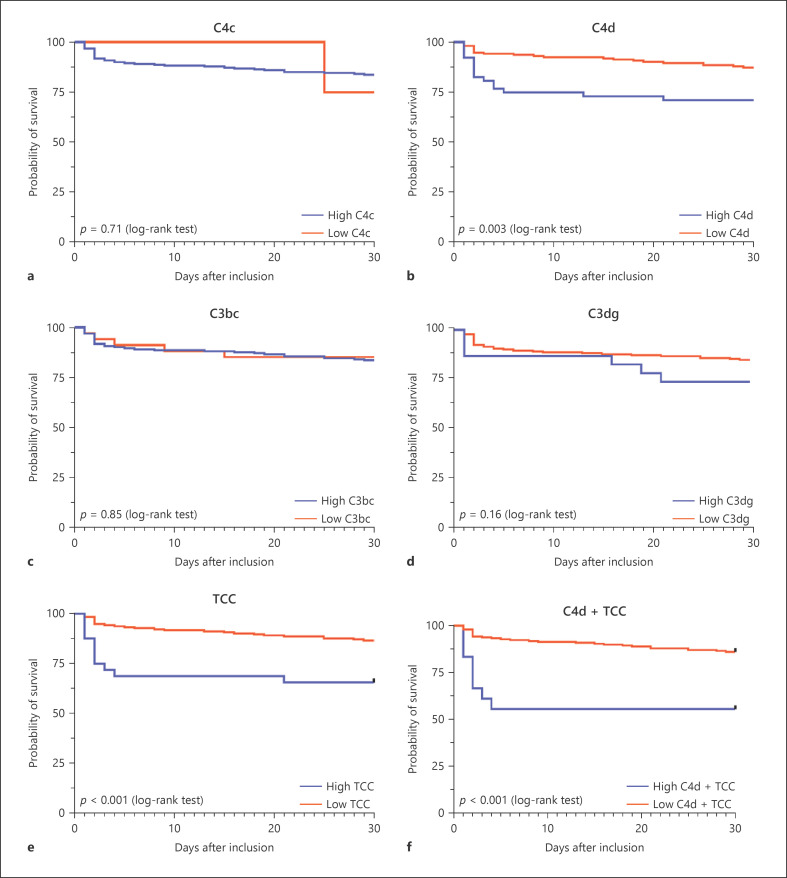
Kaplan-Meier curves of the 30-day mortality in patients with NSTI according to high and low (defined by the Youden's optimal cut-off point) baseline levels of (**a**) C4c (low *n* = 4, high *n* = 225), (**b**) C4d (low *n* = 176, high *n* = 53), (**c**) C3bc (low *n* = 34, high *n* = 195), (**d**) C3dg (low *n* = 206, high *n* = 23), (**e**) TCC (low *n* = 197, high *n* = 32), and (**f**) combined high C4d and TCC (low *n* = 211, high *n* = 18). Differences assessed by the log-rank test. NSTI, necrotizing soft-tissue infection; TCC, terminal complement complex.

**Fig. 4:**
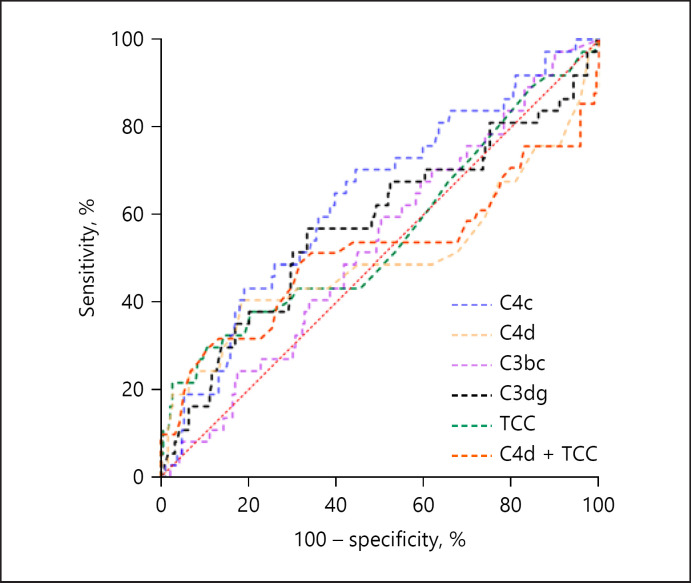
ROC curve of the 30-day mortality in patients with NSTIs for the baseline complement levels. ROC, receiver operating characteristic; NSTI, necrotizing soft-tissue infection.

Non-survivors had significantly reduced levels of C4c at baseline and day 2 and C3bc at day 1 and day 2 compared to survivors (Fig. 2). The optimal cut-off points for C3bc, C3dg, C4c, C4d, and TCC were 788 AU, 1672 AU, 180 AU, 20 ng/mL, and 31 AU, respectively (Fig. 3). In univariate Cox regression analyses, both high C4d and TCC and C4d + TCC combination showed to be associated with the 30-day mortality (Table 2). These findings were unaltered in age, sex, and comorbidity-adjusted analyses; however, when additionally adjusting for the SOFA score, only high C4d and the combination of C4d + TCC were associated with increased 30-day mortality by an HR 3.26 and HR 5.12, respectively. Prognostic accuracy according to high versus low baseline complement levels is presented in Table 3. All complement activation products demonstrated a poor ROC-AUC in predicting the 30-day mortality (Fig. 4; Table 3).

### Complement Activation and NSTI Severity

No significant differences were observed at baseline according to septic shock, receiving renal-replacement therapy, or receiving an amputation with the exception of baseline TCC, which was significantly higher in patients with septic shock than non-shock patients (Table 4). C3dg was negatively correlated with Simplified Acute Physiology Score II (SAPS II) in correlation analyses, whereas C4c demonstrated negative correlations with SAPS II, SOFA score, and blood lactate. Lastly, baseline TCC correlated with both the SOFA score and blood lactate (Table 5).

### Microbiology

A total of 58 patients had Group A *Streptococcus* in either blood and/or tissue. Of these, 50 were monomicrobial infections. Patients with Group A *Streptococcus* NSTI had significantly higher baseline TCC than in other type of microbiological NSTIs (19 AU [IQR 14–31] vs. 16 AU [IQR 11–23], *p* = 0.004). No differences were observed according to Group A *Streptococcus* NSTI on C3bc (1,928 AU [IQR 1,090–5,836] vs. 2,181 AU [IQR 1,045–5,744], *p* = 0.93), C3dg (724 AU [433–965] vs. 615 AU [IQR 399–937], *p* = 0.30), C4c (572 AU [IQR 360–746] vs. 584 AU [IQR 377–837], *p* = 0.57), and C4d (14 ng/mL [IQR 11–20] vs. 13 ng/mL [IQR 8–19], *p* = 0.10).

## Discussion

In the present study evaluating the association between various complement activation products and mortality and disease severity in patients with NSTI, we found that high levels of C4d, TCC, and the combination of these were associated with the 30-day mortality. Baseline TCC was significantly higher in patients with septic shock and correlated with disease severity. C4c and C3dg were found to be negatively correlated with disease severity.

To our knowledge, the present study is by far the largest prospectively collected cohort of patients with NSTI to give a comprehensive overview of multiple plasma complement activation products at both hospital admission and in a time-dependent manner. We evaluated different complement activation products at the C4, C3, and terminal levels. Earlier, in a smaller part of the present cohort, we have demonstrated that consumption of molecules, particularly from the lectin pathway and other downstream molecules, were associated with increased risk of death, contrasting to patients capable of maintaining high circulating levels of complement molecules [19, 20]. Of particular relevance, we have previously reported high C4 to be associated with improved survival [20], and in that context, we demonstrated in the present and larger cohort a high C4d to be associated with increased mortality. Interestingly, we have earlier found a high level of C3bc at admission to be associated with increased mortality, but this was not mirrored in the present analyses, showing no statistical association to death. Yet, in concordance with the present study, C4c does not seem to offer any prognostic properties in NSTI [20]. The complement system constitutes a vital part of the innate immune system; yet, it plays conflicting roles in the pathophysiology of sepsis [15]. In the early stages of invasive infections, the complement system establishes a central first-line of host defence, helping in containing the invading microorganisms [13]. However, exaggerated complement activation can trigger an uncontrolled inflammatory response and promote collateral tissue damage, leading to multiple organ failure and death [17, 25]. Aberrations in complement components and complement activation products have been reported in relation to various diseases, including sepsis [26]. In general, complements C4 and C3 consumption has been observed in septic patients, which is mirrored in the generation of increased levels of complement activation products, including the anaphylatoxins C4a, C3a, and C5a that are associated with severity and mortality in sepsis [27, 28, 29, 30, 31, 32]. However, anaphylatoxins are rapidly cleared from the circulation. Consequently, interpreting their systemic levels is challenging [33]. C5a is a potent pro-inflammatory peptide, and excessive production of C5a results in a series of harmful consequences, including neutrophil paralysis, apoptosis, and stimulation of pro-thrombotic activities [34]. Some activated complement components are more stable in their soluble form than others, for example, C4d and sC5b-9 (TCC) [35, 36]. C4 activation demonstrated as C4 consumption, and subsequent elevation in its activation products has been reported, concerning increased severity and mortality in septic patients [27, 37]. Furthermore, elevated TCC levels have been observed in septic patients, and the levels were found to be higher in patients with persistent septic shock than non-shock patients [38]. We investigated different activation products at the C4 and C3 level and TCC, but we only observed that C4d and TCC were increased, while the activation products C4c, C3bc, and C3dg were not increased or reduced. We interpret this as a result of the massive C4 and C3 consumption that occurred in these patients and that C4d and TCC might be more stable complement biomarkers than C4c, C3bc, and C3dg under conditions like NSTI. Our results also emphasize that choosing the proper complement activation products is critical when studying the prognostic importance of the complement system in the pathophysiology of NSTI.

We observed C4d, TCC, and a combination of both high C4d and TCC to be associated with the 30-day mortality in univariate and multivariate cox regression analyses, but after adjustment for disease severity, only high C4d and the combination of C4d and TCC were associated with an increased risk of dying within day 30 from admission. We adjusted survival analyses for age, sex, comorbidity, and disease severity as these are well-known prognostic risk factors for survival. We observed baseline TCC to be significantly elevated in patients with septic shock and those with Group A *Streptococcus* infection. This linkage may well be explained by a larger fraction of patients with this pathogen being in septic shock [2]. Of notice, none of the studied markers reached a good level of AUC-ROC in predicting the 30-day mortality, with C4c reaching the highest level of 0.64 [39]. However, AUC-ROC must be cautiously evaluated as these present the overall prognostic performance using all possible cut-off levels, including those never reaching a clinical application. Therefore, we used Youden's optimal cut-off point to estimate the best level of discriminating low versus high baseline plasma complement levels [40]. In that context, we observed all complement markers to reach markedly good NPV with C4d and TCC to reach a level of 0.87, suggesting NSTI patients with C4d below 20 ng/mL or TCC below 31 AU to have an 87% chance of surviving until day 30 after admission.

As earlier emphasized, no prognostic biomarkers have yet reached its clinical application in treatment of NSTI [8, 9, 10, 11, 12]. Yet, the present findings together with recent studies on complement activation in patients with NSTI indicate that assays on the complement system could offer important clinical information that may be used to guide the acute phase of treatment [11, 19, 20]. Bedside complement assays could be used as an easy, rapid, and reliable risk-prognosticating tool in the initial phase until more conversional disease severity tools such as SOFA or SAPS II scores can be calculated which may require 24 h of hospitalization before a valid score can be obtained, and sometimes missing values render the calculation. As the complement system seems to offer valuable prognostic information in the very first hours of NSTI, it can only be speculated if it as well can offer diagnostic properties to discriminate NSTI from less severe skin infections. This remains to be elucidated in future studies but is of high clinical relevance as no evident “red flags” exist in diagnosis of NSTI, and one of the most acknowledged diagnostic laboratory tools in NSTI − the LRINEC score − seems not robust in the recent literature [2, 41].

This study has a series of strengths. Firstly, the sample size of the study, which is by far the largest prospectively collected cohort of patients with NSTI. Secondly, we assessed both complement activation levels at admission and once daily for the next coming 3 days, thus maximizing the knowledge about complement activation in NSTI in a time-dependent manner. Thirdly, we assessed different complement activation products at different cascade levels (C4c, C4d, C3bc, C3dg, and TCC). Fourthly, no noteworthy time variation of blood sampling was present across patients as the blood was sampled by dedicated personnel according to a predefined protocol, including the specific period of daily blood sampling, subsequently followed by plasma harvesting within a short time frame. Lastly, we had a considerable high follow-up rate above 98%, increasing the validity of our findings in combination with broad inclusion criteria and only few exclusion criteria, increasing the study's generalizability. Yet, some notable limitations exist to this study, including the potential risk of missing unknown confounders in our survival analyses due to the nature of the design. Moreover, we have not published any predefined protocol on the present analyses. Lastly, multiple analyses increase the risk of chance findings.

In conclusion, we found various complement activation products to correlate with disease severity, TCC to be higher in patients with septic shock, and high baseline C4d and high C4d + TCC to be independently associated with the 30-day mortality in patients with NSTI. These findings indicate the complement system to be a valuable target for treatment interventions in NSTI, as well as a potential tool used in risk-stratification of patients with NSTI towards more individualized management.

## Statement of Ethics

This study was approved by the regional Ethics Committee (H-19016085) and the Danish Data Protection Agency (VD-2019-179). Written informed consent was obtained from all patients or their legal substitute.

## Conflict of Interest Statement

The authors do not have any conflict of interest.

## Funding Sources

The study was supported by the projects of PERMIT (Grant Number 8113-00009B) funded by Innovation Fund Denmark and EU Horizon 2020 under the frame of ERA PerMed (project 2018-151) and PERAID (Grant Number 8114-00005B) funded by Innovation Fund Denmark and NordForsk (Project No. 90456). The research leading to these results has received funding from the European Union Seventh Framework Programme (FP7/2007-2013) under the Grant agreement 305340 (INFECT project). Moreover, Copenhagen University Hospital (Rigshospitalet) provided a research grant for MH (Grant Number R167-A7352-B3897).

## Author Contributions

M.H., P.G., and O.H. contributed to study planning; P.G. contributed to laboratory analyses; M.H., P.G., and O.H. contributed to data analyses; M.H., M.M., C.B.H., P.G., and O.H. contributed to results interpretation; M.H., C.B.H., P.G., and O.H. contributed to manuscript drafting; M.H., M.M., C.B.H., P.G., and O.H. contributed to revision and approval of the final version of the manuscript.

## Data Availability Statement

The data that support the findings of this study are not publicly available but are available from the corresponding author upon reasonable request.

## Figures and Tables

**Table 1:** Patients' characteristics and outcomes

	*n* = 242
Age, years	62 (51–70)
Sex, male	144 (60)
BMI, kg/m^2^	26 (24–31)
Comorbidities	
Cardiovascular disease	110 (45)
Chronic kidney disease	17 (7)
COPD	30 (12)
Diabetes (type I and II)	68 (28)
Immune deficiency	12 (5)
Chronic liver disease	14 (6)
Malignancy	19 (8)
Peripheral vascular disease	31 (13)
Rheumatoid disease	16 (7)
No comorbidities	68 (28)
Microbiological findings^a^	
Monomicrobial infections	96 (40)
Group A *Streptococcus*	50 (52)
Polymicrobial infections	126 (52)
With the presence of Group A	
* Streptococcus*	8 (6)
No positive microbiological findings	20 (8)
Complement	
C4c, AU	572 (389–799)
C4d, ng/mL	12 (8–18)
C3bc, AU	2,090 (990–5,497)
C3dg, AU	629 (394–911)
TCC, AU	16 (11–22)
Biochemistry	
Leucocyte count, 10^9^/L	16.6 (11.1–23.4)
C-reactive protein, mg/L	226 (154–309)
Creatinine, µmol/L	109 (74–192)
Lactate, mmol/L	2.2 (1.3–3.9)
Other	
SOFA score^b^	8 (6–10)
SAPS II^c^	44 (35–55)
Septic shock upon admission^d^	114 (47)
Outcomes	
Amputation within 7 days^e^	33 (14)
RRT within 7 days	42 (17)
30-day mortality, *n* (%, 95% CI)^f^	41 (17, 13–23)

Continuous data are presented as medians (IQR) and categorical data as absolute *n* (percentage, %). Blood samples were obtained at arrival to a specialized hospital. COPD, chronic obstructive pulmonary disease; IQR, interquartile range; RRT, renal-replacement therapy.

a For further details on microbiological grouping in the present cohort can be found in Hedetoft et al. [18], 2021.

b Sequential Organ Failure Assessment (SOFA) score (day 1); data were missing for 9 (4%) patients.

c Simplified Acute Physiology Score II (SAPS II); data were missing for 9 (4%) patients.

d Septic shock was defined as lactate >2 mmol/L and the use of a vasopressor or inotrope; data were missing for 1 patient (<0.01%).

e Hundred and twelve patients with infection located to the extremities.

f Three patients were lost to the follow-up at day 90.

**Table 2:** Association between high complement levels at admission and the 30-day mortality

		Unadjusted				Adjusted analysis: age, sex, and comorbidities		Adjusted analysis: age, sex, comorbidities, and SOFA score	
	HR	95% CI	*p* value	HR	95% CI	*p* value	HR	95% CI	*p* value
C4c									
Low ≤180	Reference			Reference			Reference		
High >180	0.69	0.10–5.07	0.72	0.84	0.12–6.18	0.87	0.65	0.09–4.88	0.67
C4d									
Low ≤20		Reference			Reference			Reference	
High >20	2.26	1.36–5.06	0.004	3.08	1.57–6.01	0.001	3.26	1.56–6.81	0.002
C3bc									
Low ≤788		Reference			Reference			Reference	
High >788	1.10	0.48–2.82	0.84	1.19	0.46–3.08	0.72	1.17	0.45–3.04	0.75
C3dg									
Low ≤1,672		Reference			Reference			Reference	
High >1,672	1.84	0.77–4.41	0.17	1.99	0.82–4.85	0.13	1.82	0.68–4.86	0.23
TCC									
Low ≤31		Reference			Reference			Reference	
High >31	3.14	1.55–6.36	0.001	4.08	1.98–8.41	<0.001	1.96	0.88–4.38	0.09
C4d + TCC									
Low ≤20 & ≤31		Reference			Reference			Reference	
High >20 & >31	4.37	2.00–9.58	<0.001	5.64	2.51–12.64	<0.001	5.12	2.15–12.23	<0.001

Univariate and multivariate cox regression analyses of the 30-day mortality based on low versus high baseline complement levels according to Youden's optimal cut-off value. HR, hazard ratio; CI, confidence interval; SOFA, Sequential Organ Failure Assessment.

**Table 3:** Prognostic accuracy of complement levels on predicting the 30-day mortality

	C4c	C4d	C3bc	C3dg	TCC	C4d + TCC
Sensitivity	0.97 (0.86–1.00)	0.41 (0.25–0.58)	0.86 (0.71–0.95)	0.16 (0.06–0.32)	0.30 (0.16–0.47)	0.22 (0.10–0.38)
Specificity	0.02 (0.00–0.05)	0.80 (0.74–0.86)	0.15 (0.10–0.21)	0.91 (0.86–0.95)	0.89 (0.84–0.93)	0.95 (0.90–0.97)
PPV	0.16 (0.12–0.22)	0.29 (0.17–0.43)	0.17 (0.12–0.23)	0.26 (0.10–0.48)	0.34 (0.19–0.53)	0.44 (0.22–0.69)
NPV	0.75 (0.19–0.99)	0.87 (0.81–0.92)	0.85 (0.68–0.95)	0.85 (0.79–0.89)	0.87 (0.81–0.91)	0.86 (0.81–0.90)
AUC-ROC	0.64 (0.54–0.74)	0.50 (0.38–0.63)	0.53 (0.43–0.63)	0.58 (0.46–0.69)	0.56 (0.45–0.67)	0.52 (0.40–0.63)

Accuracy of the high complement (defined by being above the optimal cut-off point) level in predicting the 30-day mortality; C4c (low *n* = 4, high *n* = 225), C4d (low *n* = 176, high *n* = 53), C3bc (low *n* = 34, high *n* = 195), C3dg (low *n* = 206, high *n* = 23), TCC (low *n* = 197, high *n* = 32), and combined high C4d and TCC (low *n* = 211, high *n* = 18). Data are presented as fractions (95% CI). PPV, positive predictive value; NPV, negative predictive value; AUC-ROC, area under the receiver operating characteristics curve.

**Table 4:** Difference in baseline complement levels across groups

	C4c		C4d		C3bc		C3dg		TCC	
Baseline										
Shock	535 (332–762)	*p* = 0.06	14 (8–21)	*p* = 0.11	1,683 (914–4,882)	*p* = 0.07	626 (323–965)	*p* = 0.91	18 (13–29)	*p* < 0.001
Non-shock	595 (425–813)		11 (8–16)		2,434 (1,117–5,857)		623 (405–831)		14 (10–19)	
RRT	530 (343–700)	*p* = 0.26	13 (8–18)	*p* = 0.58	1,693 (980–4,856)	*p* = 0.57	512 (294–830)	*p* = 0.22	19 (13–32)	*p* = 0.17
Non-RRT	594 (397–895)		13 (9–20)		2,122 (985–6,336)		629 (406–865)		16 (13–23)	
Amputated	478 (342–714)	*p* = 0.07	13 (9–19)	*p* = 0.74	1,932 (1,070–4,440)	*p* = 0.98	550 (289–753)	*p* = 0.12	17 (14–25)	*p* = 0.24
Non-amputated	582 (401–817)		12 (8–18)		2,090 (987–5,716)		637 (403–924)		15 (11–22)	

Complement concentrations at baseline. C4d are in ng/mL, and others are presented in AU. Results presented according to patients presenting with shock at admission (114 shock vs. 128 non-shock), receiving RRT within first 7 days (42 RRT vs. 200 non-RRT) and being amputated within the first 7 days (33 amputated vs. 79 non-amputated; only patients with NSTI located to the extremities [*n* = 112] were included). Septic shock was defined as lactate >2 mmol/L and the use of a vasopressor or inotrope. Data are presented as medians (IQR). Statistical comparisons were evaluated by the Wilcoxon rank-sum test. RRT, renal-replacement therapy; IQR, interquartile range.

**Table 5:** Correlation between complement levels and markers of disease severity

	SAPS II		SOFA score		S-lactate	
	Rho	*p* value	Rho	*p* value	Rho	*p* value
C4c	–0.22	<0.001	–0.19	0.01	–0.16	0.02
C4d	–0.9	0.19	0	1	0.12	0.07
C3bc	–0.11	0.11	–0.08	0.23	–0.06	0.34
C3dg	–0.17	0.01	–0.11	0.10	–0.09	0.17
TCC	0.08	0.25	0.19	0.004	0.31	<0.001

Spearman rank correlation between severity of disease and the baseline complement level. SAPS II, Simplified Acute Physiology Score II; SOFA, Sequential Organ Failure Assessment.
